# Prevalence and Genomic Characterization of *Vibrio parahaemolyticus* Isolated from a Vast Amount of Aquatic Products in Huzhou, China

**DOI:** 10.3390/foods14142481

**Published:** 2025-07-15

**Authors:** Wei Yan, Liping Chen, Lei Ji, Rui Yuan, Fenfen Dong, Peng Zhang

**Affiliations:** Huzhou Center for Disease Control and Prevention, 999 Changxing Road, Huzhou 313000, China; yanw1024@163.com (W.Y.); lipingchen1106@hotmail.com (L.C.); jileichn@163.com (L.J.); yuanrui751@163.com (R.Y.); 15156874827@163.com (F.D.)

**Keywords:** *Vibrio parahaemolyticus*, serotype, antimicrobial susceptibility, virulence, genetic diversity

## Abstract

*Vibrio parahaemolyticus* is the leading bacterial cause of gastroenteritis associated with aquatic food consumption globally. This study aimed to determine the prevalence of *V. parahaemolyticus* in aquatic foods from Huzhou and to identify the serotypes, antimicrobial resistance, virulence factors, and genetic relatedness of the strains. A total of 306 isolates were detected from 1314 aquatic food samples from 2022 to 2024. The results indicated that the most prevalent serotypes were O1:KUT (17.0%), O2:K28 (13.7%), and O2:KUT (13.1%). Multilocus sequence typing analysis divided the 306 isolates into 175 sequence types (STs), and the predominant sequence type was ST864 (3.3%). Antimicrobial susceptibility tests showed that 2.6% of isolates were multidrug resistant. High resistance was observed to ampicillin (64.7%) and streptomycin (44.4%). A total of seven antimicrobial categories of resistance genes were identified, and the resistance gene *bla*_CARB_ was detected in all isolates. The virulence genes *tdh* and *trh* were found in 16 (5.2%) and 12 (3.9%) isolates, respectively. In addition, we observed that all the 306 *V. parahaemolyticus* isolates encode type III secretion systems 1. The phylogenomic analysis based on the whole-genome sequence revealed that the 306 isolates were divided into four clusters. Our findings broaden perspectives on *V. parahaemolyticus* genetic diversity and enhance our ability to assess the potential risks of its spread.

## 1. Introduction

Aquatic products, including fish, shrimp, and shellfish, are highly valued for their rich nutritional benefits and are enjoyed by countless consumers all over the world [1]. However, the existence of *Vibrio parahaemolyticus* (*V. parahaemolyticus*) in aquatic products can present notable health risks. *V. parahaemolyticus* is a Gram-negative halophilic bacterium found in temperate and tropical coastal areas globally [2]. As a foodborne pathogen, it causes outbreaks and sporadic cases of acute gastroenteritis associated with the ingestion of raw or undercooked contaminated food during warmer months [3,4]. In the 1990s, the first pandemic *V. parahaemolyticus* serotype of O3:K6 was identified in India [5] and subsequently spread worldwide [6,7,8]. Compared with other foodborne illnesses, infections caused by *V. parahaemolyticus* have shown a consistent rise in recent years [9]. A previous study estimated that *V. parahaemolyticus* caused approximately 35,000 human infections annually in the United States alone [10]. In other coastal countries such as Mexico, Spain, and South Korea, infection cases of *V. parahaemolyticus* have often been reported [11,12,13].

In China, *V. parahaemolyticus* became the predominant foodborne pathogen responsible for sporadic cases of foodborne disease in coastal regions, as well as the primary cause of foodborne outbreaks since the 1990s [14]. In many areas of China, the steady increase in cases of *V. Parahaemolyticus* has correlated with the pathogen’s geographic expansion. Between 2013 and 2022, the Chinese national foodborne disease surveillance network detected 23,818 laboratory-confirmed *V. parahaemolyticus* infections; moreover, this pathogen was common in the eastern coastal areas [15]. Over the past decade, Zhejiang Province, where Huzhou City is located, documented 383*V. parahaemolyticus*-associated outbreaks, with Huzhou alone accounting for 16 outbreaks affecting 301 individuals [16]. Notably, Huzhou has reported annual *V. parahaemolyticus*-related outbreaks since 2019 [17].

A previous study reported that a contamination rate of 32.2% by *V. parahaemolyticus* was observed in Chinese aquatic products [18], and the high detection rate indicated an elevated potential for *V. parahaemolyticus* infection. To date, *V. parahaemolyticus* is considered a natural inhabitant of marine environments and is frequently associated with disease outbreaks linked to seafood consumption [19]. However, in recent years, more and more studies have reported the existence of *V. parahaemolyticus* in freshwater [20,21], and consumption of freshwater foods can also lead to serious *V. parahaemolyticus* infection. *V. parahaemolyticus* exhibits varying contamination levels in freshwater foods and seafood; however, its prevalence in Huzhou remains undetermined.

In this study, between 2022 and 2024, we collected thousands of samples of seafood and freshwater foods to isolate *V. parahaemolyticus*. We profiled *V. parahaemolyticus* by assessing its prevalence, antimicrobial resistance (AMR), resistance genes, virulence factors, and phylogenomic relationships through phenotypic and genotypic approaches. These findings provide essential data for public health agencies to evaluate risks from *V. parahaemolyticus* and optimize antibiotic treatment protocols.

## 2. Materials and Methods

### 2.1. Sample Collection

A total of 1314 aquatic products were collected in Huzhou City’s three counties and two districts (Table 1) between May and September each year from 2022 to 2024, including 1102 freshwater and 212 seafood products. The collected samples encompassed diverse aquatic foods, including seafood such as fish (*n* = 126) and shellfish (*n* = 86), along with freshwater foods such as fish (*n* = 586), shrimp (*n* = 418), and snails (*n* = 98), sourced from supermarkets, restaurants, wet markets, and aquaculture sites (Table 2). The fish in the seafood category primarily included hairtail, salmon, and yellow croaker; shellfish mainly comprised crabs and clams. For freshwater food, the primary fish species were crucian carp, mandarin fish, and perch; shrimp varieties were primarily river shrimp, oriental river prawn, and crayfish; snails were predominantly river snails. Each sample was purchased in an amount of at least 500 g and individually bagged in plastic to prevent cross-contamination. All samples were immediately transported to the microbiology laboratory with in 2 h in a temperature range of 7–10 °C for further analysis. The samples were collected in compliance with the National Standards of the People’s Republic of China (GB 4789.7-2013) [22].

### 2.2. Bacterial Isolation and Identification

The isolation and identification of *V. Parahaemolyticus* were performed following the procedures of GB 4789.7-2013 [16,20]. Briefly, 25 g of each sample was inoculated into a sterile lateral filter bag containing 225 mL of sterile 3% (*w*/*v*) NaCl alkaline peptone water (Hope Bio, Qingdao, China), then the mixture was incubated at 36°C for 18 h after homogenization. The pre-enrichment solution was inoculated onto two selective media: thiosulfate-citrate-bile salt-sucrose (TCBS) agar plates (Hope Bio, Qingdao, China) and *Vibrio* chromogenic agar plates (CHROMagar, Paris, France), followed by incubation at 37°C for 18 h. Typical *V. parahaemolyticus* colonies measure 2–3 mm in diameter and appear either blue or green on TCBS agar or fuchsia on *Vibrio* chromogenic agar. At least three typical colonies of *V. parahaemolyticus* from each plate were picked and confirmed by the VITEK MS system (bioMérieux, Lyon, France). The identified *V. parahaemolyticus* isolates were stored in 3% NaCl APW medium (Hope Bio, Qingdao, China) with 20% (*v*/*v*) glycerol (Aladdin, Shanghai, China) at −80°C.

### 2.3. Serotyping

We determined the serotypes of *V. parahaemolyticus* isolates by performing slide agglutination with 11 O-specific (lipopolysaccharide) and 65 K-specific (capsule) antisera, utilizing commercially available antisera (Denka Seiken, Tokyo, Japan). Pure cultures of bacterial isolates were washed from the agar plates with 0.9%(*w*/*v*) NaCl solution. Some suspension was immediately combined with K-antigen-specific antisera to perform direct slide agglutination. The remaining suspension was autoclaved at 121 °C for 1 h, then centrifuged at 4000× *g* for 15 min. After discarding the supernatant, the pellet was washed thrice with 0.9% (*w*/*v*) NaCl solution. The final bacterial suspension underwent O-antigen agglutination testing using specific antisera, with 0.9% (*w*/*v*) NaCl solution serving as the negative control.

### 2.4. Antimicrobial Susceptibility Testing (AST)

AST of *V. parahaemolyticus* isolates was performed using the broth micro-dilution method to determine minimum inhibitory concentrations (MICs). Bacterial suspensions were adjusted to 0.5 McFarland turbidity standard, and a 10 μL suspension was inoculated into broth culture medium. Following thorough mixing, 50 μL of the inoculum was dispensed into antimicrobial susceptibility test plates, which were then sealed with breathable membranes and incubated at 36 °C for 24 h. Antimicrobial susceptibility results were interpreted using the ARIS HiQ (Thermo, Waltham, MA, USA). The 17 antimicrobials (Thermo, Waltham, MA, USA) selected were chloramphenicol (CHL, 4–32 μg/mL),trimethoprim/sulfamethoxazole (SXT, 0.5–8 μg/mL), colistin (CT, 0.25–8 μg/mL), ertapenem (ETP, 0.25–8 μg/mL), meropenem (MEM, 0.125–2 μg/mL), cefotaxime (CTX, 0.25–16 μg/mL), ceftazidime (CAZ, 0.25–16 μg/mL), ceftazidime/avibactam (CZA, 0.25/4–8/4 μg/mL), tetracycline (TET, 1–16 μg/mL), tigecycline (TIG, 0.25–8 μg/mL), ciprofloxacin (CIP, 0.015–2 μg/mL), nalidixic (NAL, 4–32 μg/mL), azithromycin (AZM, 2–64 μg/mL), amikacin (AMI, 4–64 μg/mL), streptomycin (STR, 4–32 μg/mL), ampicillin (AMP, 2–32 μg/mL), and ampicillin/sulbactam (SAM, 2–32 μg/mL).The results of AST were interpreted according to Clinical and Laboratory Standards Institute (CLSI) breakpoints (M45) [23]. Additionally, the isolates that were resistant to three or more classes of antibiotics were recorded as multidrug resistance (MDR) [24]. Blank and negative controls were established concurrently, with *Escherichia coli* ATCC 25922 employed as the reference strain.

### 2.5. PCR Detection

The hemolysin-encoding genes *tdh*, *trh*, and *tlh* were subsequently identified through PCR amplification using the *Vibrio parahaemolyticus* Triplex Nucleic Acid Detection Kit (MABSKY, Shenzhen, China). The full sequences of *tdh*, *trh*, and *tlh* genes can be found in Supplementary File S1. Fluorescent PCR reactions were prepared using a ready-to-load premix solution to which a 5 μL nucleic acid sample wasadded. The thermal cycling conditions used were as follows: 30 s at 95 °C, and 40 cycles, each cycle consisting of 5 s at 95 °C, 30 s at 60 °C, in the ABI 7500 PCR Detection System (Thermo, Waltham, USA). Fluorescence detection was performed via FAM (*tlh*), VIC (*trh*), and CY5 (*tdh*) channels for the reaction mixture. The Ct value ≤ 35 with a distinct exponential phase was interpreted as positive.

### 2.6. Whole-Genome Sequencing (WGS)

WGS was performed on all 306 *V. parahaemolyticus* isolates in this study. Genomic DNA extraction was performed on the *V. Parahaemolyticus* strains with the QIAamp DNA Mini Kit (Qiagen, Hilden, Germany) according to the manufacturer’s instructions. DNA concentration of isolates was quantified using a Qubit 4 Fluorometer (Thermo, Waltham, MA, USA). Quality-checked DNA wasstored at −80 °C in screw-cap cryovials forlong-term preservation. The whole-genome libraries were prepared utilizing the Metagenomic DNA Library Kit (Matridx, Hangzhou, China), which was used for Illumina NextSeq 550 sequencing by High-Output Reagent Cartridge v2 for 300 cycles, and 150 bp paired-end reads were generated (Illumina, San Diego, CA, USA). The sequencing depth for each sample was ≥100×, and genome coverage was ≥90%.

### 2.7. Genomic Analysis

Read quality was evaluated using FastQC (v0.11.9) and Quast (v5.1.0), requiring ≥85% of bases with Q30 quality scores, number of scaffolds ≤ 100, and ≥90% genome coverage. The reads were assembled de novo by using SPAdes (v.3.15.4). De novo assembly, which reconstructs the genome without reference dependence, offers higher accuracy for mapping short reads and ensures comprehensive characterization of highly diverse strains such as *V. parahaemolyticus*. Allele profiles and sequence types (STs) of *V. parahaemolyticus* were characterized using the standardized multilocus sequence typing (MLST) protocol with PubMLST (https://pubmlst.org/organisms/vibrio-parahaemolyticus, accessed on 29 April 2025). The antimicrobial resistance genes were analyzed by ResFinder (http://genepi.food.dtu.dk/resfinder, accessed on 8 April 2025) (v.4.7.2) with cutoffs: ≥90% identity and ≥90% coverage. The virulence factors were detected by using virulence factors of pathogenic bacteria (VFDB) database (https://www.mgc.ac.cn/cgi-bin/VFs/v5/main.cgi, accessed on 13 March 2025). Virulence factors screened against VFDB with cutoffs: ≥90% identity and ≥90% coverage. Core genome single-nucleotide polymorphisms (cgSNPs) were identified by the Snippy (v.4.4.5), with the *V. parahaemolyticus* genome GCA_000196095.1 as the reference. This analysis involved multiple steps, including “snippy-multi” and “snippy-core”, to generate core SNP alignments by using the “snippy –outdir’output’ –ref GCA_000196095.1.fna –ctgsoutput.fna” and “snippy-core –ref GCA_000196095.1.fna –prefix coresnps’output’” scripts. The core genome SNPs phylogenetic tree was generated via FastTree (v.2.1.11) by using “FastTreeMP -gtr -nt core-snps.aln>clean.core.tree.newick” script, with subsequent recombination analysis using the Gubbins (v.3.0.0) by “run_gubbins.py -p gubbins core-snps.full.aln” script. The ChiPlot was used for visualization (https://www.chiplot.online/tvbot.html, accessed on 29 April 2025) [25].

### 2.8. Statistical Analysis

Data analysis was performed in SPSS 23.0 (SPSS, Chicago, IL, USA). Differences in detection rates across sample types (seafood and freshwater food) and locations (supermarket, wet market, and restaurant) were evaluated using a chi-square test, considering *p*-values < 0.05 statistically significant.

## 3. Results

### 3.1. Prevalence and Characterization of V. parahaemolyticusin Aquatic Products

From 2022 to 2024, a total of 306 *V. parahaemolyticus* isolates were obtained from 1314 aquatic product samples collected from supermarkets, wet markets, restaurants, and aquaculture sites, yielding an overall isolation rate of 23.3% (306/1314). Among these isolates, 33.0% (101/306) were detected in 2022, 35.3% (108/306) in 2023, and 31.7% (97/306) in 2024 (Table 2). Of the total 1314 samples, 20.7% (272/1314) were collected in supermarkets, 30.0% (394/1314) in wet markets, 23.2% (305/1314) in restaurants, and 26.1% (343/1314) in aquaculture sites. Additionally, among the 306 *V. parahaemolyticus* strains, 46 were isolated from seafood samples and 260 from freshwater foods. The detection rate of *V. parahaemolyticus* was 21.7% (46/212) in seafood (27 from fish and 19 from shellfish) and 23.6% (260/1102) in freshwater foods(133 from fish, 104 from shrimp, and 23 from snails). No significant association was observed between sample types and locations in the detection rates of *V. Parahaemolyticus* (*p* = 0.352).

### 3.2. The Distribution of V. parahaemolyticus Serovars from Different Isolation Sources

**Figure 1:**
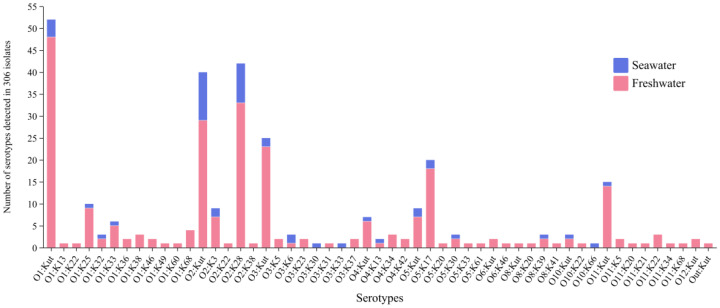
Serotype distribution of 306 *V. parahaemolyticus* isolates from aquatic products in Huzhou, collected between 2022 and 2024.

A total of 306 *V. parahaemolyticus* isolates were obtained from aquatic products in this study, and O and K antigens were identified, resulting in 11 different O groups and 29 different K types (Figure 1). Serogroups O1, O2, and O3 accounted for 70.6% (216/306), with 28.1% (86/306), 30.4% (93/306), and 12.1% (37/306)of all isolates, respectively. These 306 isolates were identified and classified into 53 distinct serotypes. Among these, 156 isolates were non-typable by K-antisera (KUT), while one isolate exhibited non-reactivity to both O- and K-antisera. The most prevalent serotypes were O1:KUT(17.0%, 52/306), O2:K28 (13.7%, 42/306), and O2:KUT (13.1%, 40/306). The seafood isolates were predominantly serotype O2:KUT(23.9%, 11/46). By contrast, the freshwater food isolates exhibited a more diverse serotype distribution, with O1:KUT being the most common at 18.5% (48/260). Among the serotypes recognized by O and K antisera, the most frequent serotypes were O2:K28 (13.7%, 42/306), O5:K17 (6.5%, 20/306), and O1:K25 (3.3%, 10/306). Notably, three strains of the O3:K6 were isolated from 306 aquatic products in this study, which were frequently linked to clinical infections in humans.

### 3.3. MLST Analysis

**Figure 2:**
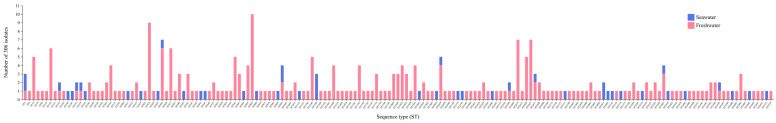
Distribution of sequence types (STs) among 306 *V. parahaemolyticus* isolates.

The MLST analysis based on seven housekeeping genes (*dnaE*, *gyrB*, *recA*, *dtdS*, *pntA*, *pyrC*, and *tnaA*) for 306 *V. parahaemolyticus* isolates showed significant molecular diversity (Figure 2). Among the 306 isolates, 175 distinct STs were identified, which highlights considerable genotypic diversity among the isolates. A total of 144 ST types were found in freshwater food strains, while 41 ST types were found in seafood strains. The most prevalent ST was ST864, accounting for 3.3% (10/306) of the isolates, followed by ST424 (2.9%, 9/306), both of which were isolated from freshwater food. For seafood, ST1250 was the most common sequence type (1.0%, 3/306). The ST864 included six different O/K serotype combinations, which were all serogroups O1, predominantly O1:KUT (40.0%, 4/10) and O1:K33 (20.0%, 2/10).

### 3.4. Antimicrobial Susceptibility of V. parahaemolyticus Isolates

In total, the 306 *V. parahaemolyticus* strains were tested for sixteen antimicrobial agents of nine different classes, including phenicols, folate pathway inhibitors, penems, cephems, tetracyclines, quinolones, macrolides, aminoglycosides, and β-lactam. As shown in Table 3, the highest rate of resistance was observed toward ampicillin (64.7%, 198/306), followed by streptomycin (44.4%, 136/306) and trimethoprim/sulfamethoxazole (3.9%, 13/306), while in lower proportion against amikacin 2.6% (8/306), nalidixic 2.0% (6/306), azithromycin 2.0% (5/306), cefotaxime 1.3% (4/306), tetracycline 1.0% (3/306), and ampicillin/sulbactam 0.6% (2/306). All *V. parahaemolyticus* strains were susceptible to ertapenem, meropenem, ceftazidime/avibactam, and tigecycline, and >90.0% of the strains were also susceptible to eight antimicrobials (chloramphenicol, trimethoprim/sulfamethoxazole, cefotaxime, ceftazidime, tetracycline, ciprofloxacin, nalidixic, and azithromycin). Additionally, a large number of strains exhibited intermediate resistance to amikacin (38.2%, 117/306), followed by streptomycin (19.3%, 59/306), ampicillin/sulbactam (14.1%, 43/306), and ampicillin (11.8%, 36/306).

In this study, eight isolates (2.6%, 8/306) were identified as MDR. The percentage of MDR strains resistant to three and four classes of antimicrobials was 2.3% (7/306) and 0.3% (1/306), respectively. One isolate was found to be resistant to four antimicrobial agents of four different classes with a resistance pattern of AMP-NAL-AZM-STR.

### 3.5. Analysis of Antimicrobial Resistance Genesand Virulence Factors

**Figure 3:**
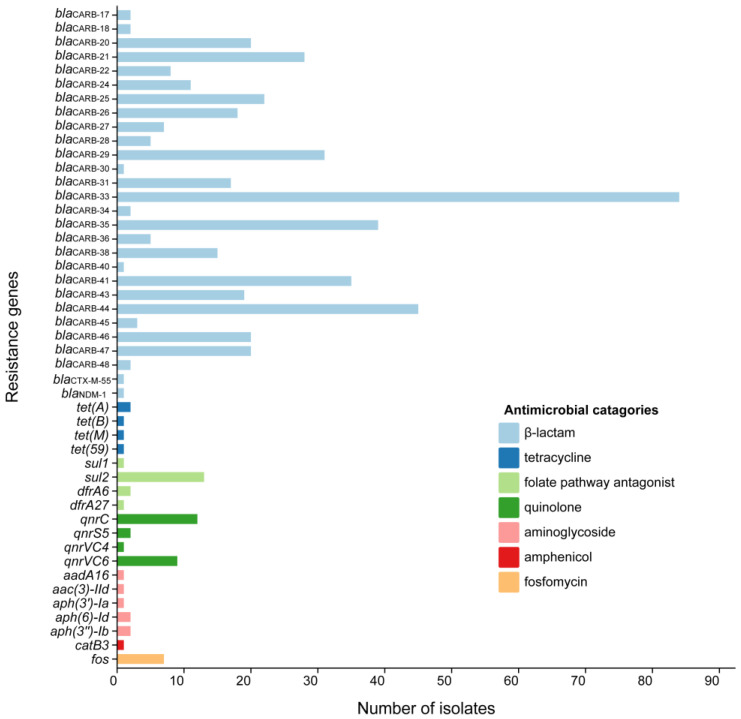
Antimicrobial resistance gene profiles of 306 *V. parahaemolyticus* strains detected with ResFinder.

After comparison and analysis using the ResFinder database, a total of seven classes of antibiotic resistance genes were identified across all 306 *V. parahaemolyticus* strains (Figure 3). All 306 *V. parahaemolyticus* isolates harbored *bla*_CARB_, with *bla*_CARB-33_ being the most prevalent (27.5%, 84/306), followed by *bla*_CARB-44_ (14.7%, 45/306) and *bla*_CARB-35_ (12.7%, 39/306). The *bla*_CARB_ gene encodes a β-lactamase enzyme, which mediates antibiotic inactivation, thereby conferring resistance to amoxicillin, ampicillin, and piperacillin. Notably, *bla*_CTX-M-55_ (conferring ESBL production) and *bla*_NDM-1_ (encoding NDM-1) were detected in two freshwater food isolates. Besides *bla*_CARB_, the most prevalent antimicrobial resistance genes were *sul2* (4.2%, 13/306), followed by *qnrC* (3.9%, 12/306), and *qnrVC6* (2.9%, 9/306). Other antibiotic resistance genes, including *fos*, *tet(A)*, and *aph(6’)-Id*, showed low prevalence (0.3–2.3%) in the aquatic product isolates.

**Figure 4:**
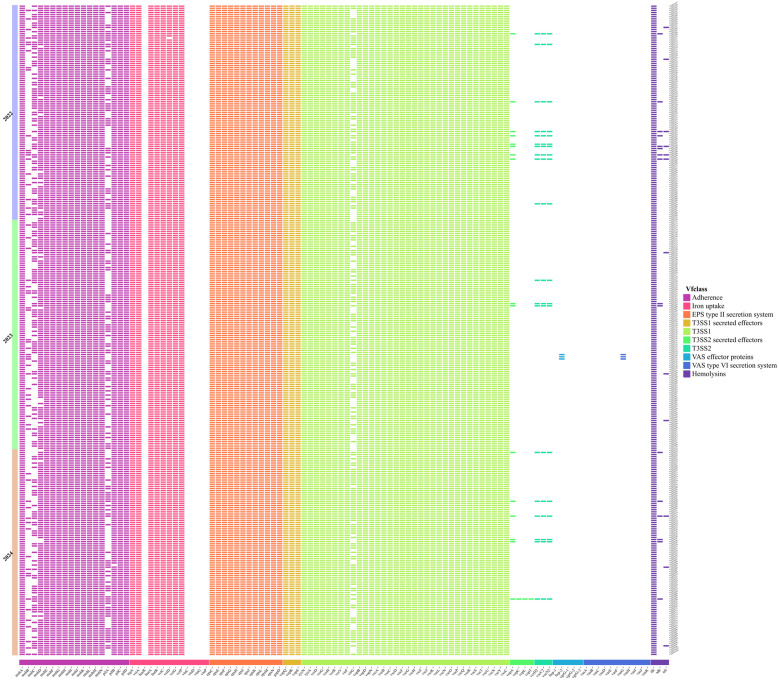
Annotation heatmap of virulence genes among 306 *V. parahaemolyticus* strains.

A total of 106 virulence genes were identified among the 306 isolates in this study (Figure 4). They were categorized into 10 classes based on the roles in pathogenesis by Vfclass, which include adherence, iron uptake, EPS type II secretion system, T3SS1 secreted effectors, T3SS1, T3SS2 secreted effectors, T3SS2, VAS effector proteins, VAS type VI secretion system, and hemolysins. All 306 isolates from aquatic products tested positive for the *tlh* gene. The *tdh* gene was detected in all 16 isolates, with 12 of these isolates exhibiting the *trh* gene. All effector proteins associated with T3SS1, including *VopQ*, *VopR*, and *VopS*, were present in every isolate. However, the detection rates of the T3SS2 effector proteins (*VopA*, *VopC*, *VopL*, and *VopT*) were relatively low, at 5.2% (16/306), 0.3% (1/306), 0.3% (1/306), and 0.3% (1/306), respectively. There was a significant difference in the detection rates between T3SS1 and T3SS2. All 306 strains carried the T3SS1 gene, whereas the carriage rate of T3SS2 was only 6.2% (19/306).

### 3.6. Phylogenetic Analysis

**Figure 5:**
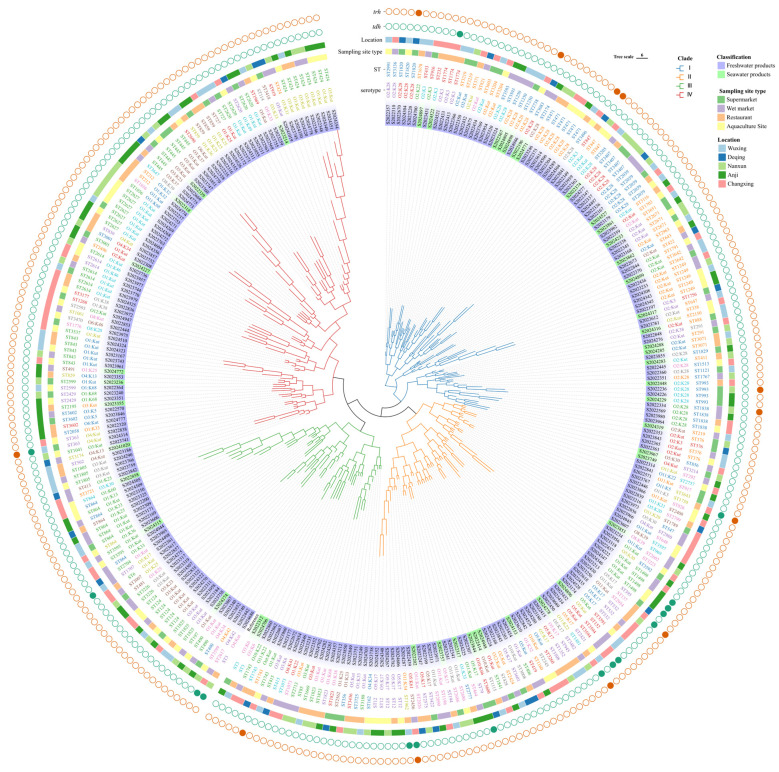
Core genome-based phylogenetic tree and distribution of classification, sampling site types, locations, serotypes, STs, and virulence factors (*tdh* and *trh*) among the 306 *V. parahaemolyticus* strains. The genome of the *V. parahaemolyticus* strain GCA_000196095.1 is used as the reference. The presence of virulence factors is shown as distinct solid color circles. Different information is distinguished by different colors.

To understand the relationships of *V. parahaemolyticus* isolated from freshwater food and seafood, a phylogenetic tree was constructed using all core SNPs extracted from the 306 isolates (Figure 5). The resulting phylogenetic tree revealed four distinct clades (I, II, III, IV). In general, all isolates from aquatic products were scattered across different clades, suggesting a high degree of genetic diversity. The variations in sampling times, sampling locations, and types of sampled food indicated the persistent presence of pathogenic *V. parahaemolyticus* in aquatic products. Nearly all O2 serotypes were distributed within clade I, whereas strains of the serogroups O1 were found across clades II, III, and IV. Phylogenetic analysis revealed genomic relatedness among isolates, as those sharing identical serotypes or STs clustered together on the phylogenetic tree. In addition, some isolates showed genetic similarity to specific clinical (human-derived) *V. parahaemolyticus* strains. As shown in Figure 5, three strains of ST3 were identified in both freshwater food and seafood, which suggests that these strains have a risk of transboundary spread to humans. Overall, the phylogenetic analysis revealed genomic relatedness among isolates from disparate geographical regions, while those from the same location showed divergent evolutionary trajectories.

## 4. Discussion

As the consumption of freshwater foods and seafood increases, new food safety concerns arise, necessitating the identification of risks posed by *V. parahaemolyticus* in aquatic products. *V. parahaemolyticus* has become one of the major causes of diarrheal disease globally; therefore, studying its prevalence and pathogenic characteristics is of great public health significance [19]. However, most studies of *V. Parahaemolyticus* have focused on clinical isolates from disease outbreaks, and research into its prevalence in aquatic products remains limited. *V. parahaemolyticus* has become the predominant bacterial pathogen in foodborne outbreaks in Huzhou, with nine outbreaks reported in the city over the past five years—averaging more than one per year [17]. This study collected 306 *V. parahaemolyticus* isolates from 1314 aquatic product samples in Huzhou, between 2022 and 2024, and assessed the prevalence, antimicrobial resistance, virulence factors, and genetic relationships of these isolates. Infections caused by *V. parahaemolyticus* show seasonal patterns, with a notable rise during warmer months, as *V. parahaemolyticus* thrives at temperatures exceeding 14°C to 19°C [26]. Rising water temperatures can increase both the distribution and incidence of *V. parahaemolyticus* [27]. To enhance detection rates, we sampled from May to September each year, when temperatures are consistently higher. In our study, the prevalence of *V. parahaemolyticus* in seafood and freshwater foods was 21.7% and 23.6%, respectively. The isolation rate in seafood was lower than reported studies from Germany (27.5%) [28] and South China (33.3%) [29], but higher than from Jilin Province (16.3%) [30]. In freshwater foods, the prevalence of *V. parahaemolyticus* was lower than that in Jiangsu Province (56.7%) [31],but higher than in Jilin Province (15.5%) [30]. The different isolation rates for *V. parahaemolyticus* were associated with multiple factors, including sampling season, sample type, hygienic conditions at terminal markets, aquaculture environments, and the choice of isolation methodology.

Serotyping has served as a cornerstone methodology in epidemiologic research and etiologic diagnosis for decades. Generally, 13 O-antigens and 71 K-antigens have been identified in *V. parahaemolyticus* [31]. Notably, a total of 11 O groups and 29 K types were identified in this study. These findings further illustrate the extensive serotype variation in environmental *V. parahaemolyticus* isolates, aligning with previous reports demonstrating broad serological diversity among environmental strains [32]. The predominant serotypes in aquatic products in this study were O1:KUT, O2:K28, and O2:KUT, which differed markedly from clinical isolates in our city (exclusively O3:K6, O10:K4, and O4:KUT) [33]. Compared to clinical isolates, environmental isolates exhibited a higher proportion of non-typable serotypes, particularly with K antisera, and most *V. parahaemolyticus* with these serotypes lacked virulence factor genes [34]. The high frequency of OUT/KUT strains among environmental isolates (51.0% in our study) reflects limitations in conventional serotyping, primarily due to antigenic diversity beyond current antiserum coverage [31].

MLST is a standard molecular typing approach that uses housekeeping gene sequences to infer bacterial population structure, diversity, and evolutionary history [35]. In this study, the MLST analysis results showed a high level of molecular diversity among the isolates collected from various food and market types in Huzhou; 306 *V. parahaemolyticus* isolates were classified into 175 STs. ST distribution had no apparent correlation with either sample types (fish, shellfish, crustacean, shrimp, and snails) or market types (supermarket, wet market, restaurant, and aquaculture site). This could be due to cross-contamination during farming, transportation, and sales of aquatic products. The predominant STs in the freshwater food isolates in this study were ST864 and ST424, while the study from Jiangsu Province demonstrated that the most common STs wereST1750 and ST1703 [21]. Similar to seafood, ST1250 predominated in this study, while ST2601 was the primary ST in Jiangsu Province [21]. That further confirmed the high diversity of *V. parahaemolyticus* isolates in aquatic products. Notably, some STs such as ST3, ST445, and ST993 have been identified in both freshwater food and seafood, indicating potential transmission of *V. parahaemolyticus* across freshwater food and seafood. Moreover, the presence of ST3 in aquatic products merited significant attention; ST3 was the predominant sequence type among clinical strains [17,36], which was found to carry genes for *tdh*, T3SS, T6SS, and virulence genomic islands [30]. While ST3 predominated in human clinical cases (79.4%) in Huzhou [17], our detection of ST3 in local food samples at a lower prevalence (1.0%) indicates potential foodborne transmission of this pathogenic sequence type. Therefore, continuous surveillance and molecular characterization of *V. parahaemolyticus* are critical to better understand its evolutionary dynamics and potential health impacts.

Previous studies had reported that *V. parahaemolyticus* was susceptible to a wide range of antibiotics [37,38]. In this study, all *V. parahaemolyticus* isolates were susceptible to ETP, MEM, CZA, and TIG; the majority were also susceptible to CHL (99.0%), SXT (96.1%), CTX (98.7%), CAZ (99.3%), TET (97.0%), CIP (99.6%), NAL (98.0%), and AZM (98.0%), which was consistent with previous reports [36,39]. Current guidelines recommend doxycycline or quinolones for treating diarrhea associated with *V. parahaemolyticus* infection [40]. Our findings demonstrate that quinolones remain clinically effective as first-line therapy for gastroenteritis caused by *V. parahaemolyticus*. Consistent with Zheng et al. [30], ampicillin resistance prevailed among *V. parahaemolyticus* isolates in this study, with most strains exhibiting resistance (64.7%). In addition, international surveillance data revealed ampicillin resistance rates of 40–100% in *V. Parahaemolyticus* isolates, consistent with our findings [38,41]. Notably, a significantly elevated STR resistance rate (44.4%) was documented in this study, exceeding values reported in prior surveillance [42]. These results can be ascribed to the variable use of antimicrobials in different regions. Consequently, antimicrobial selection for clinical and aquacultural applications must be guided by region-specific resistance surveillance data. Multidrug-resistant *V. Parahaemolyticus* emergence represents a grave epidemiological concern, as it limits the available antibiotics for treatment. Fortunately, multidrug resistance was detected in only 8 isolates (2.6%) among the 306 *V. parahaemolyticus* strains under antimicrobial surveillance of Huzhou. However, implementing stringent surveillance and control across the entire food production-supply chain is critical.

All isolates from aquatic products carried the *bla*_CARB_ gene, and most were resistant to ampicillin, indicating that ampicillin resistance was significantly correlated with the presence of the *bla*_CARB_ gene. However, no streptomycin-resistant genes were detected in the 306 genomes, potentially due to short-read sequencing’s limitations in resolving mobile genetic elements [43]. Notably, genes associated with ESBL production were detected in isolates. While the *bla*_CTX-M-55_ variants identified here do not necessarily confer phenotypic resistance (such as ceftazidime) under current conditions, their presence raises concerns about the potential accumulation of resistance determinants in environmental reservoirs. Beyond the *bla*_CARB_ gene, detected antibiotic resistance genes in *V. parahaemolyticus* frequently involved *sul2* (sulfonamide resistance) and *qnr* (quinolone resistance), aligning with prior studies [44]. Some isolates carried resistance genes but remained phenotypically susceptible to antibiotics, which may be attributed to the genes being low-expressed or conferring low resistance levels that do not exceed the interpretive breakpoint [45]. In general, antibiotic-resistant genes could not fully explain their resistance phenotypes.

Thermostable direct hemolysin (TDH) and TDH-related hemolysin (TRH) are the primary virulence factors of *V. parahaemolyticus*, as they can form channels in the membranes of intestinal epithelial cells and induce inflammatory gastroenteritis rapidly [45]. Thus, *tdh/trh* gene detection provided a critical indicator of virulence potential in *V. parahaemolyticus*. It has been demonstrated that clinical *V. parahaemolyticus* strains typically carry the virulence genes *tdh* and/or *trh*, whereas these genes are rarely found in environmental isolates (such as those from aquatic products) [46]. In this study, *tdh*+ and *trh* + *V. parahaemolyticus* isolates accounted for 5.2% (16/306) and 3.9% (12/306) of all isolates, respectively, and were considered pathogenic strains. A similarly low detection rate has been observed globally, 4.3% (9/208) of *V. Parahaemolyticus* strains from Mexican oysters carried *tdh*+ and/or *trh*+ genes [32]; 3% of pathogenic (*tdh*+) strains were reported in the USA [47]; and Japanese seafood isolates showed *tdh*+/*trh*−, *tdh*−/*trh*+, and *tdh*+/*trh*+ rates of 5.4%, 12.2%, and 4.1%, respectively [48].In China, *V. parahaemolyticus* isolated from aquatic foods harbored *tdh/trh* genes at frequencies ranging from 5.5% to 10.3% [36]. These findings indicate that *V. parahaemolyticus* producing TDH and/or TRH was not highly prevalent; however, its wide distribution and clustering in certain regions may pose a potential threat to aquatic food safety.

Besides *tdh* and *trh*, other virulence factors, such as the Type III secretion system (T3SS), play key roles in mediating enterotoxicity and cytotoxicity. The T3SS mediates the translocation of bacterial effector proteins directly into eukaryotic cells to exert their functions [49]. In our study, T3SS1 effector proteins (*VopQ*, *VopR*, and *VopS*) and T3SS2 effector proteins (*VopA*) were present in 100.0% (306/306) and 5.2% (16/306) of the isolates, respectively. Genomic analysis confirmed the coexistence of the *tdh* and T3SS2-associated *vopA* genes in *V. parahaemolyticus*, aligning with prior reports [30,36].

A phylogenetic tree based on core genome analysis showed that the 306 *V. parahaemolyticus* isolates were divided into four distinct clades (I, II, III, and IV). As shown in Figure 5, four clades included isolates from freshwater food and seafood, with no obvious clustering in the phylogenetic tree. Extensive genomic polymorphism enhanced *V. parahaemolyticus*’ adaptation to dynamic environments [50]. Previous research had identified cross-contamination of *V. parahaemolyticus* between seafood and freshwater food as a risk during the transport and sale chain [17,21]. Strains harboring the *tdh* gene, which were exclusively isolated from freshwater foods and found in all four clades, indicate a potential human infection risk associated with freshwater food. All three ST3 strains (isolated from freshwater foods and seafood) were classified into clade III and occupied neighboring positions on the phylogenetic tree. Overall, the phylogenomic analysis revealed that *V. parahaemolyticus* isolates in the environment of Huzhou were genetically diverse. Therefore, continuous surveillance of *V. Parahaemolyticus* evolutionary trends is essential to reduce infection incidence.

This study has several limitations. First, due to budget constraints, only second-generation draft genome sequencing was performed on the isolates, precluding comprehensive characterization of their complete genetic profiles. Second, we acknowledge the lack of experimental validation regarding the functional implications of identified antimicrobial resistance and virulence genes. Specifically, investigating the pathogenicity of food-derived *tdh*-carrying *V. parahaemolyticus* isolates will constitute a priority focus for future research.

## 5. Conclusions

This study analyzed the prevalence, serotypes, antimicrobial resistance, virulence factors, molecular typing characteristics, and genetic relationships of 306 *V. parahaemolyticus* strains recovered from aquatic products in Huzhou. The study identified high contamination rates and antibiotic resistance, alongside a low occurrence of virulence-associated genes. The high genetic diversity and virulence potential of *V. parahaemolyticus* strains isolated from aquatic foods strengthen the public health risk assessment of this pathogen. The presented data provide a basis for sanitary authorities and aquatic products producers to evaluate food safety and establish effective control strategies against *V. parahaemolyticus* infections caused by consuming aquatic foods.

## Figures and Tables

**Table 1:** Locations and types of Sampling sites evaluated in the present study.

SamplingSites	Locations	Coordinate	
		Latitude	Longitude
Wet market	Wuxing	30°50′19″ N	120°9′13″ E
Supermarket	Wuxing	30°54′30″ N	120°6′3″ E
Restaurant	Wuxing	30°51′52″ N	120°5′43″ E
Aquaculture site	Wuxing	30°53′45″ N	120°12′6″ E
Wet market	Nanxun	30°51′38″ N	120°25′9″ E
Supermarket	Nanxun	30°52′10″ N	120°26′14″ E
Restaurant	Nanxun	30°52′19″ N	120°25′3″ E
Aquaculture site	Nanxun	30°43′17″ N	120°15′39″ E
Wet market	Deqing	30°32′33″ N	119°57′56″ E
Supermarket	Deqing	30°31′43″ N	119°58′7″ E
Restaurant	Deqing	30°32′7″ N	119°58′36″ E
Wet market	Changxing	30°59′2″ N	119°54′54″ E
Supermarket	Changxing	31°0′13″ N	119°54′21″ E
Restaurant	Changxing	31°1′39″ N	119°54′49″ E
Aquaculture site	Changxing	31°5′49″ N	119°52′50″ E
Wet market	Anji	30°38′0″ N	119°41′37″ E
Supermarket	Anji	30°37′42″ N	119°41′15″ E
Restaurant	Anji	30°38′39″ N	119°41′13″ E
Aquaculture site	Anji	30°47′29″ N	119°39′46″ E

**Table 2:** Prevalence of *V. parahaemolyticus* isolated from aquatic products in Huzhou (2022–2024).

Location	Seafood		Freshwater Food			Total
	Fish	Shellfish	Fish	Shrimp	Snails	
Supermarket	20.0%(6/30)	18.1%(4/22)	21.7%(23/106)	23.2%(19/82)	/	21.7%(52/240)
Wet market	22.2%(12/54)	23.7%(9/38)	25.0%(47/188)	27.4%(34/124)	21.2%(11/52)	24.8%(113/456)
Restaurant	21.4%(9/42)	23.1%(6/26)	23.3%(20/86)	26.7%(20/75)	26.1%(12/46)	24.4%(67/275)
Aquaculture site	/	/	20.9%(43/206)	22.6%(31/137)	/	21.6%(74/343)
Total	21.7%(46/212)		23.6%(260/1102)			23.3% (306/1314)

**Table 3:** Antimicrobial resistance of 306*V. parahaemolyticus* isolates.

Antimicrobial Catagories	Antimicrobials	Breakpoints (MIC, µg/mL)			Number of Isolates (Percentage)		
		Susceptible	Intermediate	Resistant	Susceptible	Intermediate	Resistant
Phenicols	CHL	≤8	16	≥32	303 (99.0)	3 (1.0)	0 (0)
Folate pathway inhibitors	SXT	≤2/38	–	≥4/76	294 (96.1)	0 (0)	12 (3.9)
Penems	ETP	≤0.5	1	≥2	306 (100.0)	0 (0)	0 (0)
	MEM	≤1	2	≥4	306 (100.0)	0 (0)	0 (0)
Cephems	CTX	≤1	2	≥4	302 (98.7)	0 (0)	4 (1.3)
	CAZ	≤4	8	≥16	304 (99.3)	2 (0.7)	0 (0)
	CZA	≤8/4	–	≥16/4	306 (100.0)	0 (0)	0 (0)
Tetracyclines	TET	≤4	8	≥16	297 (97.0)	6 (2.0)	3 (1.0)
	TIG	≤0.5	–	>0.5	306 (100.0)	0 (0)	0 (0)
Quinolones	CIP	≤0.06	0.12–0.5	≥1	305 (99.6)	1 (0.4)	0 (0)
	NAL	≤16	–	≥32	300 (98.0)	0 (0)	6 (2.0)
Macrolides	AZM	≤16	–	≥32	301 (98.0)	0 (0)	5 (2.0)
Aminoglycosides	AMI	≤16	32	≥32	181 (51.2)	117 (38.2)	8 (2.6)
	STR	≤8	16	≥32	111 (36.3)	59 (19.3)	136 (44.4)
β-lactam	AMP	≤8	16	≥32	72 (23.5)	36 (11.8)	198 (64.7)
	SAM	≤8/4	16/8	≥32/16	261 (85.3)	43 (14.1)	2 (0.6)

## Data Availability

The original contributions presented in this study are included in the article. Further inquiries can be directed to the corresponding author or first author. Generated sequencing data are available under NCBI BioProject PRJNA1225868.

## Supplementary Materials

The following supporting information can be downloaded at: https://www.mdpi.com/article/10.3390/foods14142481/s1. File S1: The full sequences of *tdh*, *trh*, and *tlh*.
