# Supplementary material for: Prevalence and Genomic Characterization of Vibrio parahaemolyticus Isolated from a Vast Amount of Aquatic Products in Huzhou, China
Source: Foods. 2025 Jul 15;14(14):2481. doi: 10.3390/foods14142481 (PMC12295969; doi:10.3390/foods14142481)
Supplement: Supplementary file 1 [file foods-14-02481-s001.zip › foods-3733998-supplementary.pdf]

The full sequences of *tdh*:
“ATGAAGTACCGATATTTTGCAAAAAAATCATTTTTATTTATATCCATGTTGGCTGCA
TTCAAAACATTTGCCTTTGAGCTTCCATCTGTCCCTTTTCCTGCCCCCGGTTCTGAT
GAGATATTGTTTGTTGTTCGAGATACAACTTTTAATACCAATGCACCGGTCAATGTA
GAGGTCTCTGACTTTTGGACAAACCGTAATGTAAAAAGAAAACCGTACAAAGAT
GTTTATGGTCAATCAGTATTCACAACGTCAGGTACTAAATGGCTGACATCCTACAT
GACTGTGAACATTAATGATAAAGACTATACAATGGCAGCGGTGTCTGGCTATAAG
CACGGTCATTCTGCTGTGTTCGTAAAATCAGATCAAGTACAGCTTCAACATTCCTA
TGATTCTGTAGCTAACTTTGTTGGTGAAGATGAAGATTCTATTCCAAGTAAAATGTA
TTTGGATGAAACTCCAGAATATTTTGTTAATGTAGAAGCATATGAGAGTGGTAGTG
GTAATATATTGGTAATGTGTATATCCAACAAAGAATCGTTTTTTGAATGTAAACATC
AACAATAA”

The full sequences of *trh*:
“ATGAAACTAAAACTCTACTTTGCCTTCAGTTTGCTATTGGCTTCGATATTTTCAGTA
TCTAAATCATTCGCGATTGACCTGCCATCCATACCTTTTCCTTCTCCAGGTTCGGAT
GAGCTACTATTTGTCGTTAGAAATACAACAATAAAAACTGAATCACCAGTTAACG
CAATCGTTGATGACTACTGGACAAACCGAAACATAAAACGAAAACCATATAAAA
GCGTTCACGGTCAATCTATTTTCACGACTTCAGGCTCAAAATGGTTAAGCGCCTAT
ATGACGGTAAATATTAATGGAAATAACTACACAATGGCTGCTCTTTCTGGCTATAA
AGATGGCCTTTCAACGGTCTTCACAAAATCAGAAAAAACAAGCCTAAATCAGAA
CTATTCTTCTGTTAGTGATTTCGTTGGTGAGAATGAAGAATCATTGCCAAGTGTAA
CGTATTTGGATGAAACGCCAGAATATTTCGTCAATGTCGAAGCATATGAGAGCGG
AAATGGGCATATGTTTGTTATGTGTATTTCCAATAAATCATCATTTGATGAATGTATG
TCACAAAATTAA”

The full sequences of *tlh*:
“ATGATGAAAAAAACAATCACACTATTAACTGCATTACTCCCGCTTGCTTCTGCAG
TTGCCGAAGAGCCAACCTTATCACCAGAAATGGTTTCAGCGTCTGAAGTGATCAG
CACGCAAGAAAACCAAACCTATACCTATGTTCGCTGTTGGTATCGCACCAGCTAC
TCGAAAGATGATCCAGCGACCGATTGGGAATGGGCAAAAAACGAAGATGGTAGC
TACTTCACCATTGACGGCTACTGGTGGAGCTCCGTTTCATTTAAAAACATGTTCTA
CACCAACACGTCGCAAAACGTTATCCGTCAGCGTTGTGAAGCAACATTAGATTTG
GCGAACGAGAACGCAGACATTACGTTCTTCGCCGCTGACAATCGCTTCTCATACA
ACCACACGATCTGGAGCAACGACGCAGCAATGCAGCCAGATCAAATCAACAAA
GTGGTTGCACTCGGTGACAGCTTGTCTGATACAGGCAACATCTTTAACGCATCAC
AATGGCGCTTCCCTAACCCGAACAGCTGGTTCTTAGGTCACTTCTCCAACGGTTTT
GTTTGGACAGAATACATTGCCAAAGCGAAGAACCTTCCGCTCTACAACTGGGCAG
TTGGCGGCGCGGCTGG”
